# Self-report of worse back pain among teachers at state schools in Minas Gerais during the COVID-19 pandemic

**DOI:** 10.47626/1679-4435-2022-998

**Published:** 2023-11-24

**Authors:** Maria Alice Moura Soares, João Matheus de Almeida Ravnjak, Rosângela Ramos Veloso Silva, Desirée Sant’Ana Haikal, Rose Elizabeth Cabral Barbosa, Lucineia Pinho

**Affiliations:** 1 Curso de Medicina, Universidade Estadual de Montes Claros (Unimontes), Montes Claros, MG, Brazil; 2 Curso de Medicina, Centro Universitário Faculdades Integradas Pitágoras de Montes Claros, Montes Claros, MG, Brazil; 3 Programa de Pós-Graduação em Cuidado Primário em Saúde, Unimontes, Montes Claros, MG, Brazil; 4 Programa de Pós-graduação em Ciências da Saúde, Unimontes, Montes Claros, MG, Brazil

**Keywords:** back pain, musculoskeletal pain, symptom flare up, cumulative trauma disorders, dor nas costas, dor musculoesquelética, exacerbação dos sintomas, transtornos traumáticos cumulativos

## Abstract

**Introduction:**

One of the consequences of halting face-to-face educational activities during the COVID-19 pandemic was worse back pain.

**Objectives:**

To evaluate worse back pain in teachers working in elementary state schools in Montes Claros, MG.

**Methods:**

This is a websurvey-type epidemiological survey using an on-line questionnaire to assess sociodemographic characteristics, working conditions, health condition, and behaviors during the pandemic. Poisson regression was performed, with robust variance.

**Results:**

15,641 teachers were included, and 35.4% reported worse back pain during the pandemic. It was found that the prevalence of a worse condition was higher among women (prevalence ratio = 1.15), between 40 and 49 years old (prevalence ratio = 1.14), teaching for more than 11 years (prevalence ratio = 1.11; 1.19), working more than 21 hours (PR = 1.05; 1.11), with difficulty to work remotely (prevalence ratio = 1.16), with poor quality of life (prevalence ratio = 1.30) or not (prevalence ratio = 0.84), obese (prevalence ratio = 1.07), sad or depressed (prevalence ratio = 1.21), anxious or nervous (prevalence ratio = 1.57), consuming alcoholic beverage (prevalence ratio = 1.16), with poor dietary habits (prevalence ratio = 1.07), more screen time (prevalence ratio = 1.24), sedentary lifestyle (prevalence ratio = 1.13), and social distancing (prevalence ratio = 1.08).

**Conclusions:**

The pandemic worsened back pain in teachers, demonstrating a need for addressing the issue, aiming at improving the quality of life of these professionals.

## Abstract (pt)

**Introdução:**

Uma das consequências da suspensão das atividades presenciais educacionais durante a pandemia da covid-19 foi o aumento da dor nas costas.

**Objetivos:**

Avaliar o aumento da dor nas costas em professores, em atividade, na educação básica de escolas estaduais de Montes Claros, Minas Gerais.

**Métodos:**

Trata-se de inquérito epidemiológico do tipo *websurvey*, através de questionário *on-line*, para avaliação das características sociodemográficas, condições de trabalho, situação de saúde e comportamentos durante a pandemia. Foi realizada regressão de Poisson, com variância robusta.

**Resultados:**

Participaram 15.641 professores, sendo que 35,4% relataram piora da dor nas costas durante a pandemia. Houve prevalência da piora do quadro nos seguintes grupos: sexo feminino (razão de prevalência = 1,15), idade entre 40 e 49 anos (razão de prevalência = 1,14), acima de 11 anos na docência (razão de prevalência = 1,11; 1,19), jornada de trabalho superior a 21 horas (razão de prevalência = 1,05; 1,11), dificuldade no trabalho remoto (razão de prevalência = 1,16), qualidade de vida com piora (razão de prevalência = 1,30) ou não (razão de prevalência = 0,84), obesos (razão de prevalência = 1,07), tristes ou deprimidos (razão de prevalência = 1,21), ansiosos ou nervosos (razão de prevalência = 1,57), que consumiam bebidas alcóolicas (razão de prevalência = 1,16), com pior padrão alimentar (razão de prevalência = 1,07), tempo de tela aumentado (razão de prevalência = 1,24), ausência de atividades (razão de prevalência = 1,13) e adesão total ao distanciamento social (razão de prevalência = 1,08).

**Conclusões:**

O período pandêmico trouxe um agravo na dorsalgia em professores, o que demonstra uma necessidade de atenção à questão, visando uma melhoria na qualidade de vida desses profissionais.

## INTRODUCTION

Chronic back pain is a major cause of sick leave in the working class and the most common musculoskeletal condition among outpatient complaints.^1^ This pain has different classifications and etiologies. However, a common factor in all etiologies is muscle and joint overload related to stress arising from the occupational profile of some workers, such as teachers.^2^

During the COVID-19 pandemic, social isolation and halting of classroom educational activities in public and private schools were measures implemented to prevent the disease. Thus, students and teachers had to adapt to remote teaching, a change that affected working conditions, causing professionals to be exposed to inadequate working environment, spending hours looking at screens, and an increased workload.^3,4^

Due to these circumstances, teachers complained of exacerbated stress, associated with fear and anxiety due to the distance and imminent risk of contamination.^5,6^ Thus, these conditions, combined with changes in work and leisure habits, affected the physical and mental health of teachers. As a result, musculoskeletal pain (especially back pain), a sedentary lifestyle, and chronic diseases, such as obesity, have been increasingly observed in this population.^7^

Therefore, as the condition represents a burden on the health system and has a high incidence in the working class, analyzing the impacts of changes in work profile of teachers, as was the case during the COVID-19 pandemic, it is important to clarify the actual conditions these professionals have faced. Therefore, this study assessed the effects of the COVID-19 pandemic on worse back pain among teachers, considering sociodemographic characteristics, teaching-related issues, health condition, and behaviors during the pandemic.

## METHODS

### STUDY DESIGN

This study is part of the core research project ProfSMoc - Etapa Minas Covid “Condições de saúde e trabalho dos professores da educação básica da rede estadual de ensino de Minas Gerais na pandemia do COVID-19” (Health and work conditions of basic education teachers in the state education system of Minas Gerais in the COVID-19 pandemic). Teachers and researchers from the State University of Montes Claros (Unimontes) conducted an epidemiological websurvey with support from the State Department of Education of Minas Gerais (SEE-MG).

### POPULATION AND SAMPLE

The population included about 90,000 teachers from the state public school system of Minas Gerais. Primary education in Brazil includes pre-school, primary and secondary education. In Minas Gerais, six regional centers are divided in 47 teaching superintendent departments. This study used an infinite populations formula to calculate the sample size. A minimum participation of 2,564 teachers was estimated, considering a 50% prevalence rate of events, a 95% confidence interval (95%CI), 3% error, def = 2, and a 20% increase for possible losses.

The inclusion criteria were to be teaching in 2020; to work in preschool, elementary and/or secondary school; to be working in a state school; and to accept to participate in the study voluntarily. Retired teachers, those who answered “no” when asked if they agreed to participate in the study, and teachers working in a position other than teaching were excluded. No restrictions were placed on teachers on sick leave or other reasons to participate in the study.

### STUDY VARIABLES AND DATA COLLECTION

Teachers were provided with an online questionnaire on the Google Forms^®^ platform. The link to the form was sent by SEE-MG to the institutional e-mail of all teachers in the state. A reCAPTCHA with image tests was used to avoid filling out the form using robots. Data were collected between August 20 and September 11, 2020.

The dependent variable in this study was back pain, which was assessed by answering the question “Have changes in your usual activities affected your back pain during the pandemic?”. Teachers were classified according to the presence (increased a little and increased a lot) or absence (no back pain, decreased, and remained the same) of back pain.

The independent variables were organized in subject groups:

- Sociodemographic characteristics: sex (male or female); age group (up to 39 years, 40 to 49 years, or 50 years and older); marital status (affirms or denies having a partner); have children or not; family income (one to three minimum wages, four to six minimum wages, seven minimum wages or more).
- Working conditions as a teacher: time working as a teacher (up to 10 years, 11 to 20 years, 21 years or more); weekly working hours (up to 20 hours, 21 to 40 hours, 40 hours or more); difficulty to teaching remotely (none or little, moderate, or much).
- Health condition and behaviors during the pandemic: quality of life (improved, unchanged, worsened); increased tobacco consumption (no, yes); increased alcohol consumption (no, yes); diet (better dietary habits, poor dietary habits); increased screen time (no, yes); obesity (no, yes); physical activity (no, yes); often feeling sad or depressed (no, yes); often feeling anxious or nervous (no, yes); social distancing (no or partially, totally).

### DATA ANALYSIS

The variables analyzed were described as absolute and relative frequencies. The prevalence of the analyzed behaviors was estimated using their 95%CI. The association between the dependent and independent variables was assessed using the crude and adjusted prevalence ratio (PR), estimated by Poisson regression with robust variance. Bivariate analyses were initially performed, and variables with a descriptive level (p value) ≤ 0.20 were selected for the multivariate model (adjusted analysis), with a significance level of 0.05. All statistical analyses were performed using Stata, version 13.0.

### ETHICAL ISSUES

The study complied with Resolution 466/12 of the Brazilian National Health Council/Ministry of Health, which deals with studies with human beings. The project was submitted to the Unimontes Research Ethics Committee and was approved with a substantiated opinion No. 4.200.389/2020.

## RESULTS

The study included 15,641 teachers, 81.8% women. The mean age was 43 years (± 9.4), mean time working as a teacher was 14.8 years (± 9.5). Among the participants, 35.4% reported worse back pain during the pandemic. The other characteristics, related to sociodemographic variables, to teaching, and to habits and behaviors during the pandemic are presented in Table 1.

**Table 1:** Distribution of the study population according to sociodemographic characteristics, characteristics of the job as a teacher, and habits and behaviors during the pandemic, ProfSMoc - Etapa Minas Covid, State of Minas Gerais, 2020 (n = 15,641).

Variables	n	%
Sociodemographic characteristics		
Sex		
Male	2,824	18.1
Female	12,817	81.9
Age (years)		
Up to 39	6,447	41.2
40 a 49	8,793	56.2
50 or more	401	2.6
Marital status		
No partner	5,188	33.2
Partnered	10,453	66.8
Do you have any children?		
No	4,291	27.4
Yes	11,350	72.6
Household income (minimum wages)		
1 to 3	8,195	52.4
4 to 6	6,122	39.1
7 or more	1,324	8.5
Characteristics of the job as a teacher		
Time working as a teacher (years)		
Up to 10	5,941	38.0
11 to 20	5,788	37.0
21 or more	3,911	25.0
Weekly working hours		
Up to 20	4,433	28.4
21 to 40	9,735	62.2
41 or more	1,473	9.4
Difficulty to teaching remotely		
None or little	5,656	36.2
Moderate or much	9,985	63.8
Health condition and behaviors during the pandemic		
Your quality of life		
Improved	1,359	8.7
Had no changes	3,789	24.2
Worsened	10,493	67.1
Increased tobacco use		
No	15,239	97.4
Yes	402	2.6
Increased alcohol consumption		
No	14,482	92.6
Yes	1,159	7.4
Diet (eating habits)		
Better	7,737	49.5
Worse	7,904	50.5
Increased screen time		
No	1,001	6.4
Yes	14,640	93.6
Obesity^*^		
No	11,706	76.1
Yes	3,679	23.9
Do you do physical exercise?		
Yes	8,373	53.5
No	7,268	46.5
Do you often feel sad or depressed?		
No	8,377	53.6
Yes	7,264	46.4
Do you often feel anxious or nervous?		
No	6,514	41.7
Yes	9,127	58.3
Social distancing		
No or partially	3,155	20.2
Totally	12,486	79.8

* Pregnant women were excluded from the analysis (n = 256).

The results of the bivariate analysis between the outcome worse back pain during the pandemic and the sociodemographic, occupational, behavioral, and health variables among teachers during the pandemic are presented in Table 2. Variables that showed a significant association at the 0.20 level with the outcome worse back pain during the pandemic were selected for multivariate analysis.

**Table 2:** Bivariate analysis, worse back pain, ProfSMoc - Etapa Minas Covid, State of Minas Gerais, 2020 (n = 15,641).

Variables	p-value (%)	Crude PR (95%CI)	p-value
Sociodemographic characteristics			
Sex			
Male	27.9	1.00	
Female	37.1	1.33 (1.25-1.42)	0.000
Age (years)			
Up to 39	31.8	1.00	
40 a 49	38.4	1.21 (1.15-1.26)	0.000
50 or more	28.4	0.89 (0.76-1.05)	0.167
Marital status			
No partner	35.0	1.00	
Partnered	35.7	1.02 (0.97-1.07)	0.397
Do you have any children?			
No	33.1	1.00	
Yes	36.3	1.10 (1.05-1.15)	0.000
Household income (minimum wages)			
1 to 3	33.9	1.00	
4 to 6	36.8	1.09 (1.04-1.14)	0.000
7 or more	39.1	1.15 (1.07-1.24)	0.000
Characteristics of the job as a teacher			
Time working as a teacher (years)			
Up to 10	30.7	1.00	
11 to 20	36.5	1.19 (1.13-1.25)	0.000
21 or more	41.0	1.34 (1.27-1.41)	0.000
Weekly working hours			
Up to 20	31.7	1.00	
21 to 40	36.3	1.15 (1.09-1.21)	0.000
41 or more	40.7	1.28 (1.19-1.38)	0.000
Difficulty to teaching remotely			
None or little	27.6	1.00	
Moderate or much	39.8	1.44 (1.37-1.51)	0.000
Health condition and behaviors during the pandemic			
Your quality of life			
Improved	25.7	1.00	
Had no changes	20.8	0.81 (0.73-0.90)	0.000
Worsened	42.0	1.63 (1.49-1.79)	0.000
Increased tobacco use			
No	35.2	1.00	
Yes	46.0	1.31 (1.18-1.46)	0.000
Increased alcohol consumption			
No	34.3	1.00	
Yes	49.5	1.44 (1.36-1.54)	0.000
Diet (eating habits)			
Better	32.7	1.00	
Worse	38.1	1.16 (1.12-1.21)	0.000
Increased screen time			
No	23.4	1.00	
Yes	36.3	1.55 (1.38-1.74)	0.000
Obesity^*^			
No	34.1	1.00	
Yes	39.8	1.17 (1.11-1.22)	0.000
Do you do physical exercise?			
Yes	30.9	1.00	
No	40.6	1.31 (1.26-1.37)	0.000
Do you often feel sad or depressed?			
No	24.7	1.00	
Yes	47.8	1.94 (1.86-2.03)	0.000
Do you often feel anxious or nervous?			
No	21.0	1.00	
Yes	45.7	2.17 (2.06-2.29)	0.000
Social distancing			
No or partially	30.4	1.00	
Completely	36.7	1.21 (1.14-1.28)	0.000

* Pregnant women were excluded from the analysis (n = 256).

95%CI = confidence interval; PR = prevalence ratio.

The multivariate model, presented in Table 3, showed that the prevalence of worse back pain among teachers was higher among women (PR = 1.15) and in the age group between 40 and 49 years (PR = 1.14). As for the characteristics of the job as a teacher, the highest prevalence of worse back pain was observed among teachers who reported have been working for 11 to 20 years (PR = 1.11) and 21 years or more (PR = 1.19), working 21 to 40 hours per week (PR = 1.05) and 41 hours or more (PR = 1.11), and have moderate/much difficulty to teach remotely (PR = 1.16).

**Table 3:** Multivariate analysis, worse back pain, ProfSMoc - Etapa Minas Covid, State of Minas Gerais, 2020 (n = 15,641).

Variables	Adjusted PR (95%CI)	p-value
Sociodemographic characteristics		
Sex		
Male	1.00	
Female	1.15 (1.08-1.23)	0.000
Age (years)		
Up to 39	1.00	
40 a 49	1.14 (1.09-1.20)	0.000
50 or more	1.02 (0.87-1.19)	0.810
Characteristics of the job as a teacher		
Time working as a teacher (years)		
Up to 10	1.00	
11 to 20	1.11 (1.05-1.17)	0.000
21 or more	1.19 (1.11-1.26)	0.000
Weekly working hours		
Up to 20	1.00	
21 to 40	1.05 (1.00-1.11)	0.033
41 or more	1.11 (1.03-1.19)	0.006
Difficulty to teaching remotely		
None or little	1.00	
Moderate or much	1.16 (1.11-1.22)	0.000
Health condition and behaviors during the pandemic		
Your quality of life		
Improved	1.00	
Had no changes	0.84 (0.75-0.93)	0.001
Worsened	1.30 (1.18-1.43)	0.000
Increased alcohol consumption		
No	1.00	
Yes	1.16 (1.09-1.24)	0.000
Diet (eating habits)		
Better	1.00	
Worse	1.07 (1.02-1.12)	0.002
Increased screen time		
No	1.00	
Yes	1.24 (1.11-1.38)	0.000
Obesity^*^		
No	1.00	
Yes	1.07 (1.02-1.12)	0.003
Do you do physical exercise?		
Yes	1.00	
No	1.13 (1.08-1.18)	0.000
Do you often feel sad or depressed?		
No	1.00	
Yes	1.21 (0.15-1.29)	0.000
Do you often feel anxious or nervous?		
No	1.00	
Yes	1.57 (1.46-1.68)	0.000
Social distancing		
No or partially	1.00	
Completely	1.08 (1.02-1.14)	0.011

95% CI = confidence interval; PR = prevalence ratio.

* Pregnant women were excluded from the analysis (n = 256).

As for the health condition of teachers during the pandemic, a higher prevalence of worse back pain was found among those who reported that their quality of life had remained unchanged (PR = 0.84) or worsened (PR = 1.30), were obese (PR = 1.07), often felt sad or depressed (PR = 1.21), and anxious or nervous (PR = 1.57). As for behaviors and lifestyle habits, we observed an association between those with increased consumption of alcohol (PR = 1.16), poor dietary habits (PR = 1.07), increased screen time (PR = 1.24), sedentary lifestyle (PR = 1.13), and total social distancing (PR = 1.08).

## DISCUSSION

This study showed that some teachers from Minas Gerais state school system had significant worse back pain during the pandemic, an outcome associated with psychological conditions, sociodemographic characteristics, and lifestyle.

A study with 782 teachers working from home during the pandemic in Slovakia reported 74% of neck pain, 67% of low back pain, and 60% of pain in any other parts of the back. Among the major causes associated with these pains were stress, sedentary lifestyle, and poor work conditions.^2^ Previous data, published with the same population of this study, showed that 58% of teachers reported worse back pain due to changes during the pandemic; risk factors for back pain were being a woman, longer working hours, psychological issues, and lifestyle.^8^

This study found a higher prevalence of worse back pain among women. This result was expected, since women represented more than 81% of the population included in the study. In addition, according to the literature, back pain is more prevalent among women, which can be explained based on women-related hormonal, reproductive, and body composition issues.^9^ Some authors, when evaluating the effects of stress on the lifestyle of women during the COVID-19 pandemic, associated a higher risk with women overloaded with housework and home care.^10^

In relation to age group, older age was found to be among the factors associated with worse back pain. Aging causes changes in intervertebral discs, damaging them, causing pain and limiting human capacity to work and move.^11^ A study with teachers in Chile before and during the pandemic found that teachers over 45 years of age reported greater impairment of physical body functions than teachers below this age group, which indirectly reinforces the findings of our study.^12^

Data from a previous study showed that the respondents associated poorer quality of life with worse back pain. Another study with teachers during the pandemic reported poorer quality of life in several age groups. However, among those aged over 45 years, worsening was predominantly associated with limitations related to physical functions.^12^

Health condition can be greatly influenced by dietary habits, and this study demonstrated that an unhealthy diet caused, to some extent, worse back pain among teachers during the pandemic. Considering the time and the outcome analyzed, the overall diet of the population has been noticeably modified, leading to a higher consumption of hypercaloric, processed foods, with high fat content, which affects the physical and mental health of the individual.^13^

Increased screen time is harmful in several ways to human beings. The equation that shows that increasing screen time indicates more time in an inadequate posture and more time inactive contributes to increasing sedentary lifestyle and, thus, to worse pain, as this study revealed. According to a study conducted in India with 629 workers working remotely in the educational system, 60.8% reported musculoskeletal pain or discomfort and 71% associated discomfort directly with working remotely, combined with more screen time.^14^

Physical inactivity was found to worsen back pain. Therefore, a decrease in this habit during the pandemic resulted in a worse overall quality of life, combined with other variables, such as poorer dietary habits and increased screen time,^7,13,15^ which result from the effects of an increased sedentary lifestyle during the pandemic due to the protective measures taken.

A study with Chilean teachers before and during the pandemic showed that teachers’ ability to effectively maintain their mental health worsened, and working remotely was a major factor affecting psychosocial health and causing physical exhaustion due to burnout and stress.^12^ This scenario may have contributed to worsen back pain in this population. According to previous studies investigating risk factors for back pain, with emphasis on psychological issues, psychopathologies have a strong influence on the ability to perceive and identify pain, corroborating an often harmful distortion of pain that may not be compatible with the pathological amplitude of pain.^16-18^

This study showed that alcohol consumption was positively associated with back pain. However, the association of alcohol consumption with chronic back pain in previous studies is weak and inconsistent, and the relationship is only strong in cases of alcohol-dependent individuals.^19^

Obesity has also been found as a risk factor for back pain; however, this association is contradictory.^20^ A previous study supports this association and claims that obesity increases the mechanical load of the back, causing greater strain on the structures of the lumbar spine during daily activities, thus causing pain. In addition, another explanation is that obesity can cause chronic systemic inflammation, as this condition increases cytokines and acute phase reactants and activates pro-inflammatory pathways, thus causing pain.^21^

Prolonged exposure to the teaching activity was another risk factor found. Previous studies found that, even before the pandemic, teachers were subjected to precarious working conditions, with several demands that favored the increase of back pain.^22^ In the pandemic, with the transition of teachers to online education, studies have shown an increase in the intensity and duration of back pain and related limitations, since teleworking aggravated the precariousness of teaching and negatively modified the working conditions.^23^

The changes in the characteristics of teaching jobs has also affected the pace and quality of teaching throughout the world.^24^ The results showed that teachers had a significant difficulty to teleworking, demonstrating that factors such as logistics difficulties for some students and teachers, as illustrated in the remote structure of the services used to teach classes, also influenced the outcome, leading to a distortion of the effectiveness of the knowledge construction process during the pandemic.^5^ This difficulty is seen in the increased physical and mental stress of these teachers, which, as is well known, worsens the perception of pain and, consequently, worsens back pain.^25^

Social distancing was also found to be a risk factor for back pain, according to a study conducted with professors in Minas Gerais, Brazil, who had their mental health and lifestyle affected due to stress, anxiety, and other factors. Thus, the changes caused their quality of life to worsen, which may corroborate, for example, the worsening of back pain.^26^

Some possible limitations of this cross-sectional study are relevant, as it has the capacity to provide only a snapshot of the exposure and the effect studied. Therefore, data on exposure and outcome were collected only momentarily, thus temporal assessment between cause and effect is not permitted.

As the process of studying and working using digital tools is projected to grow in the coming years, these results warn against an urgent need to create strategies to improve these workers’ health. Consequently, this study is important because it categorizes which impacts will be magnified in the long run as new digital culture are created, if they are not addressed properly. These physical and psychological issues negatively affect the health of workers globally, especially teachers, leading to reduced work capacity, poorer health and leisure, thus a higher incidence of illness.

## CONCLUSIONS

This study found that the pandemic worsened chronic back pain among teachers in the State of Minas Gerais. Therefore, attention to these professionals is necessary, reinforcing the indispensable practice of physical exercises, improvement in dietary habits, reduction in screen time, and professional psychological counseling. In addition, a need to create collaborative networks to help teachers get the technical support they need to prepare to work remotely is clear.
